# A Yeast-Based Repurposing Approach for the Treatment of Mitochondrial DNA Depletion Syndromes Led to the Identification of Molecules Able to Modulate the dNTP Pool

**DOI:** 10.3390/ijms222212223

**Published:** 2021-11-12

**Authors:** Giulia di Punzio, Micol Gilberti, Enrico Baruffini, Tiziana Lodi, Claudia Donnini, Cristina Dallabona

**Affiliations:** Department of Chemistry Life Sciences and Environmental Sustainability, University of Parma, Parco Area delle Scienze 11/A, 43124 Parma, Italy; giulia.dipunzio@unipr.it (G.d.P.); micol.gilberti@unipr.it (M.G.); enrico.baruffini@unipr.it (E.B.); tiziana.lodi@unipr.it (T.L.); cristina.dallabona@unipr.it (C.D.)

**Keywords:** yeast, mitochondrial DNA depletion syndromes (MDS), drug repurposing, MPV17, SYM1, POLG, MIP1, RRM2B, RNR2, mitochondrial dNTP pool

## Abstract

Mitochondrial DNA depletion syndromes (MDS) are clinically heterogenous and often severe diseases, characterized by a reduction of the number of copies of mitochondrial DNA (mtDNA) in affected tissues. In the context of MDS, yeast has proved to be both an excellent model for the study of the mechanisms underlying mitochondrial pathologies and for the discovery of new therapies via high-throughput assays. Among the several genes involved in MDS, it has been shown that recessive mutations in MPV17 cause a hepatocerebral form of MDS and Navajo neurohepatopathy. MPV17 encodes a non selective channel in the inner mitochondrial membrane, but its physiological role and the nature of its cargo remains elusive. In this study we identify ten drugs active against MPV17 disorder, modelled in yeast using the homologous gene *SYM1*. All ten of the identified molecules cause a concomitant increase of both the mitochondrial deoxyribonucleoside triphosphate (mtdNTP) pool and mtDNA stability, which suggests that the reduced availability of DNA synthesis precursors is the cause for the mtDNA deletion and depletion associated with Sym1 deficiency. We finally evaluated the effect of these molecules on mtDNA stability in two other MDS yeast models, extending the potential use of these drugs to a wider range of MDS patients.

## 1. Introduction

Mitochondrial DNA depletion syndromes (MDS) are clinically heterogenous and often severe diseases. These syndromes are characterized by a reduction, in one or several tissues, of the mitochondrial DNA (mtDNA) copy number. The decrease of this biomarker of mitochondrial function can be caused by mutations in mitochondrial or nuclear genes [1,2].

Recessive mutations in MPV17, encoding a protein of 176 amino acids [3] located in the inner mitochondrial membrane (IMM) [4], have been associated with a hepatocerebral form of MDS and Navajo neurohepatopathy [4,5]. MPV17 protein and its mouse (Mpv17), zebrafish (tra/Mpv17) and *Saccharomyces cerevisie* (Sym1) orthologs show a high degree of conservation [6]. MPV17 takes part within a high molecular weight complex [7,8] and forms a non selective channel in the IMM affecting mitochondrial membrane potential, reactive oxygen species (ROS) production and cristae formation [7,9,10,11]. However, the physiological role of the channel and the nature of the cargo remain elusive. A role of this protein in mtDNA maintenance has been suggested in humans, rats and yeast by the observation that mtDNA instability occurs in the absence of Sym1/MPV17, but the connection remains unclear. The reduction of dGTP and dTTP nucleotide pools, observed in mitochondria of rat tissues and fibroblasts derived from patients with mutations in the MPV17 gene, suggest that mitochondrial dNTP insufficiency is the principal cause of mtDNA depletion [12]. Additionally, the ortholog gene *tra*/*mpv17* has been related to nucleotide metabolism in zebrafish [13,14], whereas in *S. cerevisiae,* the ortholog *SYM1* has been related to the homeostatic control of tricarboxylic acid cycle (TCA) intermediates [7]. 

Therapeutic strategies with potential general applicability to several mitochondrial diseases have been proposed (reviewed in [15,16]). An effective strategy against the deleterious role of cristae disruption in mitochondrial pathogenic mechanisms has been successfully exploited in a mouse [11], thanks to a moderate overexpression of Opa1 [17], a master regulator of shape and function of mitochondrial cristae.

However, despite the advances in molecular and biochemical methodologies leading to a better understanding of the etiology and mechanisms of mitochondrial disorders, there are still no satisfactory therapies available for these diseases [15]. The treatments for mitochondrial diseases are limited to symptomatic relief and do not significantly alter the course of the diseases [18]. 

Yeast has proved to be not only an excellent model for the study of the mechanisms underlying mitochondrial pathology [19,20,21,22,23] but also for the discovery of new therapies. High-throughput yeast-based assays have been successfully used by different groups [24,25] to identify drugs active against several human mitochondrial disorders [20,26,27,28,29,30,31]. The possibility of screening collections of thousands of drugs approved by the Food and Drug Administration (FDA) allows a drug repurposing approach, thus speeding up the drug discovery process by identifying new clinical uses of already available molecules [32,33]. In this study, we report the results of a phenotypic screening of 1018 compounds from the Selleck Chemicals FDA-approved library by exploiting a yeast mutant strain carrying the *sym1^R51W^* mutation. Ten drugs that were able to rescue the oxidative growth defective phenotype of the *sym1* mutant were also able to rescue the mtDNA instability phenotype. We further demonstrated that *sym1∆* mitochondria display a shortage in the mitochondrial dNTP pool and that the pool is rescued by the addition of the identified molecules, thus establishing that the decrease in mitochondrial dNTPs is the cause of mtDNA depletion in Sym1 deficiency. Some of these molecules were also effective in other MDS yeast models, in particular those characterized by mutations in *MIP1* and *RNR2*, orthologs of the human genes POLG and RRM2B respectively, potentially expanding their application to a wider range of MDS patients.

## 2. Results

### 2.1. Identification of Molecules Preventing the Oxidative Phosphorylation Defect Associated with the Sym1 Mutation

Thanks to the high degree of conservation between human MPV17 protein and its yeast homolog Sym1, several *sym1* mutant alleles have been previously constructed by introducing human MPV17 mutations in the corresponding position of the *SYM1* gene [4,34].

**Figure 1:**
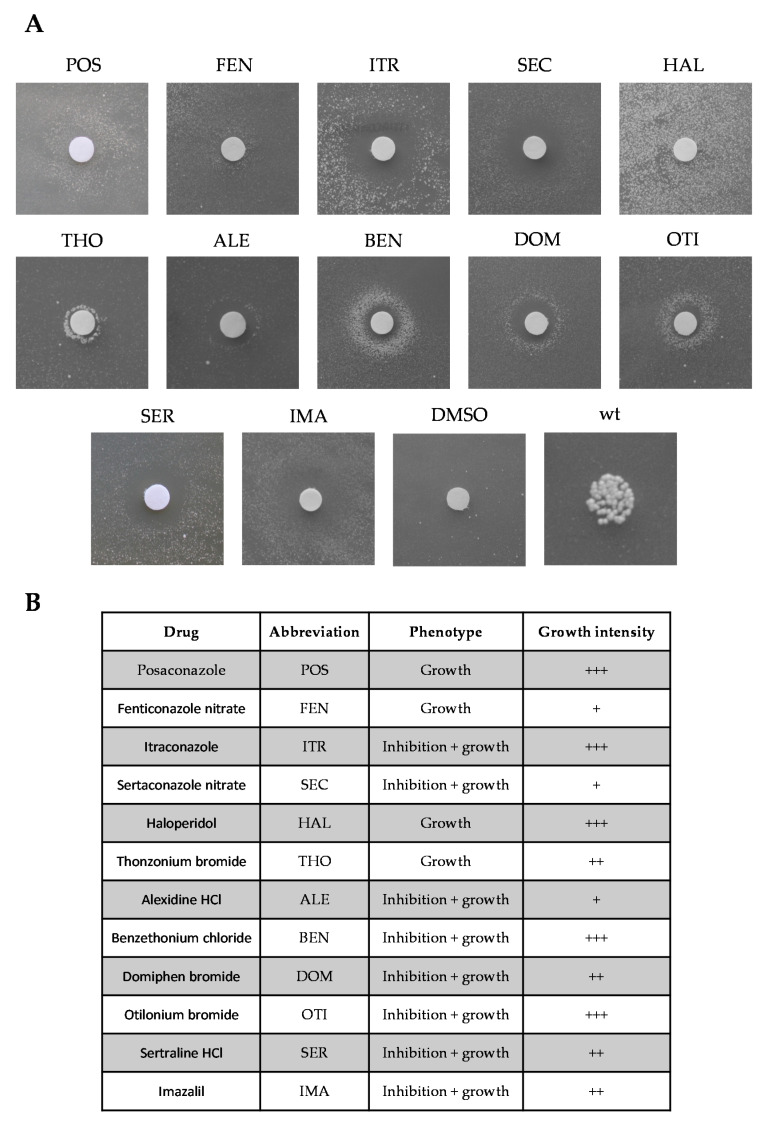
Identification of drugs rescuing the defective oxidative growth of the s*ym1^R51W^* mutant strain. (**A**) 1.5 × 10^5^ cells were spread on a 12 × 12 cm square plate containing solid YP medium supplemented with 4% ethanol. Sterile filters were placed onto the solid medium surface and 2.5 μL of the different drugs were loaded at a concentration of 10 mM. Active compounds were identified after 72–96 h incubation at 37 °C by the halo of growth around the filters. One filter was loaded with the same amount of dimethyl sulfoxide (DMSO), the solvent in which the molecules were solubilized. As a positive growth control, the wild-type strain was used as described in Materials and Methods. (**B**) Rescuing effects on the oxidative growth: +++ strong effect; ++ medium effect; + mild effect were visually evaluated by the intensity of the growth halo. Each drug was used at a concentration of 10 mM.

To search for active compounds against MPV17-related diseases, we performed a yeast-based pharmacological screening, exploiting the temperature-sensitive (ts) yeast mutant harboring the R51W recessive variant, equivalent to the human MPV17 R50W pathological variant. This strain (s*ym1^R51W^*) exhibits limited growth at 37 °C in yeast peptone (YP) medium supplemented with 2% ethanol [4], an oxidative carbon source that requires functional mitochondria to be used. This growth defective phenotype made it possible to search for molecules that restore this growth defect. A total of 1018 compounds belonging to the Selleck Chemicals chemical library were tested for their ability to rescue the oxidative phosphorylation (OXPHOS) growth defect. After the identification of the best experimental condition (4% ethanol, 1.5 × 10^5^ cell/plate and 37 °C), the screening was performed in two steps. Potentially active molecules were initially identified after 6 d of incubation at 37 °C by the appearance of a halo of growth around the filters soaked with drugs. Positive drugs were tested again in a dedicated plate, and 11 confirmed their beneficial effect (Figure 1). A further beneficial molecule, Imazalil (Figure 1), was identified among six compounds from the Tebu-Bio or Prestwick chemical libraries, previously identified as beneficial in another MDS yeast model (personal observation).

The identified molecules showed different intensities of growth rescue (Figure 1B). Most of them had a toxic effect at higher concentrations, as indicated by the formation of a halo of inhibition near the filter and a halo of growth far from the filter (phenotype: inhibition + growth). Others had a rescuing effect at the maximum concentration tested (10 mM), as indicated by the appearance of a halo of growth near the filter (phenotype: growth).

The target enzymes of the identified molecules in yeast and mammals are reported in Table 1. Interestingly, 6 of the 12 identified molecules (Posaconazole, Fenticonazole nitrate, Itraconazole, Sertaconazole nitrate, Imazalil and Haloperidol) have the same target in yeast, the ergosterol pathway. Of the five molecules that have the same target enzyme, lanosterol 14-α-demethylase (Table 1), we subsequently characterized only three, i.e., Posaconazole, Fenticonazole nitrate and Imazalil. 

We then analyzed whether the beneficial effect of the identified molecules was due to an improvement of Sym1 functionality or a bypass of Sym1 function. For this reason, we evaluated the capacity of the molecules to rescue the defective OXPHOS growth phenotype of the *sym1Δ* strain. All the tested molecules were able to rescue the oxidative growth defect of the null mutant, suggesting that they exert their beneficial effect through a bypass mechanism (Supplementary Figure S1).

### 2.2. Characterization of the Effects of the Identified Molecules on mtDNA Maintenance

**Figure 2:**
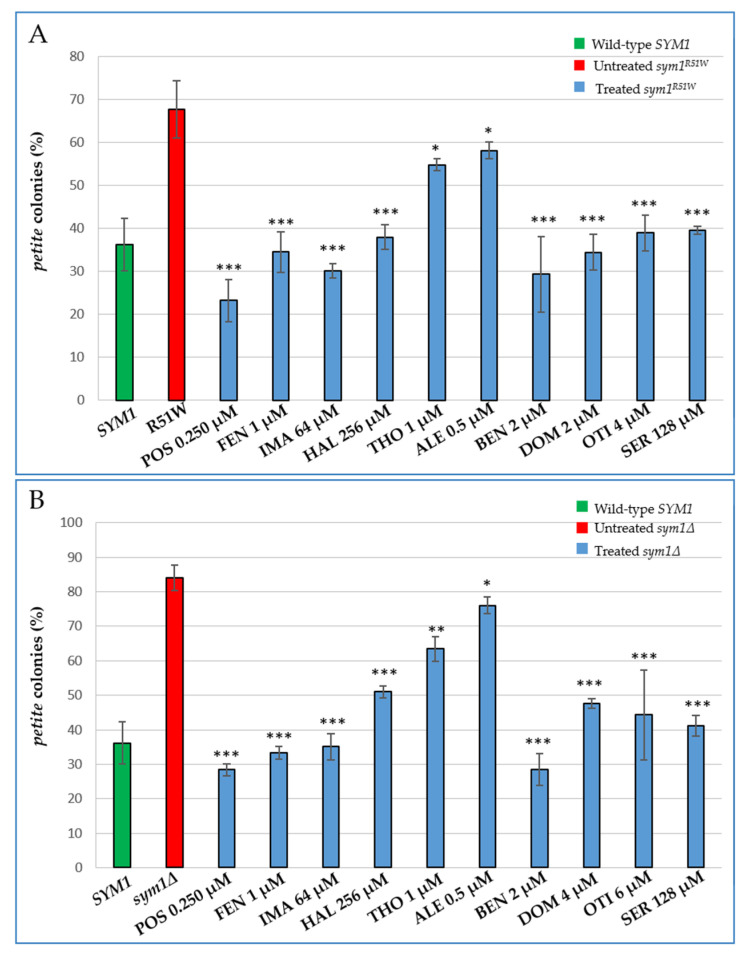
Effect of the identified drugs on *petite* frequency of the *sym1^R51W^* (**A**) and *sym1∆* mutant strains (**B**). Cells were grown at 37 °C in synthetic complete (SC) medium supplemented with 2% glucose and 2% ethanol. More than 4000 colonies/strain were scored. Values are represented as the mean of at least three independent experiments ± standard deviation (SD). Statistical analyses were performed using one-way analysis of variance (ANOVA) followed by a Bonferroni’s post hoc test comparing treated (blue bars) versus the untreated mutant (red bar) in which the compound vehicle DMSO was added: * *p* < 0.05; ** *p* < 0.01; *** *p* < 0.001.

The *sym1∆* strain and all the strains carrying mutations equivalent to the pathological alleles present in humans studied so far, are characterized by a significant increase of *petite* mutants [4,34]. These mutants arise spontaneously in the presence of large deletions or a depletion of mtDNA which gives rise to mitochondrial respiratory deficient cells [35]. To further explore the effects of the active compounds, we evaluated their impact on mtDNA stability in the *sym1^R51W^* mutant strain by measuring the *petite* frequency after treatment with different subminimal inhibitory concentrations (sub-MICs) of the molecules. The addition of all the molecules significantly improved the mitochondrial genome stability of *sym1^R51W^* cells (Figure 2A). With the exception of Thonzonium bromide and Alexidine HCl, which showed a mild reduction of the *petite* frequency of about 20% and 15%, respectively, all the other molecules displayed a strong effect. They reduced the *petite* percentage by about 50% or more vs. the mutant and with values equivalent or smaller than those of the wild-type.

As all the selected molecules were able to rescue the oxidative growth defect in the absence of the Sym1 protein, we also evaluated their effect on the mtDNA instability of the *sym1Δ* strain (Figure 2B). A remarkable reduction of the instability of mtDNA after supplementation with all the identified drugs was observed. The results for Posaconazole, Fenticonazole nitrate, Imazalil and Benzethonium chloride showed them to be the most active molecules, bringing the percentage of *petite* colonies to a level equal or even lower than the wild-type. 

To exclude the possibility that the beneficial effect observed was due to a selective induction of the *petite* mutants’ mortality, a fitness test was performed growing both BY4741 *rho^+^* (with wild-type mtDNA) and BY4741 *rho^0^* (devoid of mtDNA) cells together, mixed in an equal amount in the presence or absence of the molecules (Supplementary Figure S2). Since the percentage of *rho^0^* was similar with and without the tested drug molecules, the reduction of *petite* percentage was ascribed to a positive effect of the drugs on mitochondrial genome stability.

### 2.3. Characterization of the Effects of the Identified Molecules on the Mitochondrial dNTP Pool

The limited availability of precursors for DNA synthesis has been demonstrated in MPV17 deficient mice, zebrafish and fibroblasts, suggesting that the depletion of mtDNA in cells lacking MPV17 is due to a shortage of mitochondrial dNTPs. We therefore proceeded by determining the levels of mitochondrial dNTPs in the Sym1 deficient yeast. Mitochondrial dNTPs were extracted from cells grown under conditions in which the *petite* frequency was similar between the *sym1Δ* mutant and the wild-type *SYM1* strain (0.6% glucose and 2% ethanol for 24 h at 37 °C) (Supplementary Figure S3). It is a critical requirement to compare the two strains in a condition where they show an equal mtDNA stability and the mutant does not exhibit mtDNA loss. Indeed, in the absence of Sym1 a large percentage of cells become *rho^0^*. In *rho^0^* cells derived from a wild-type strain, the amount of dTTP is drastically reduced compared to *rho*^+^ cells (Supplementary Figure S4). 

**Figure 3:**
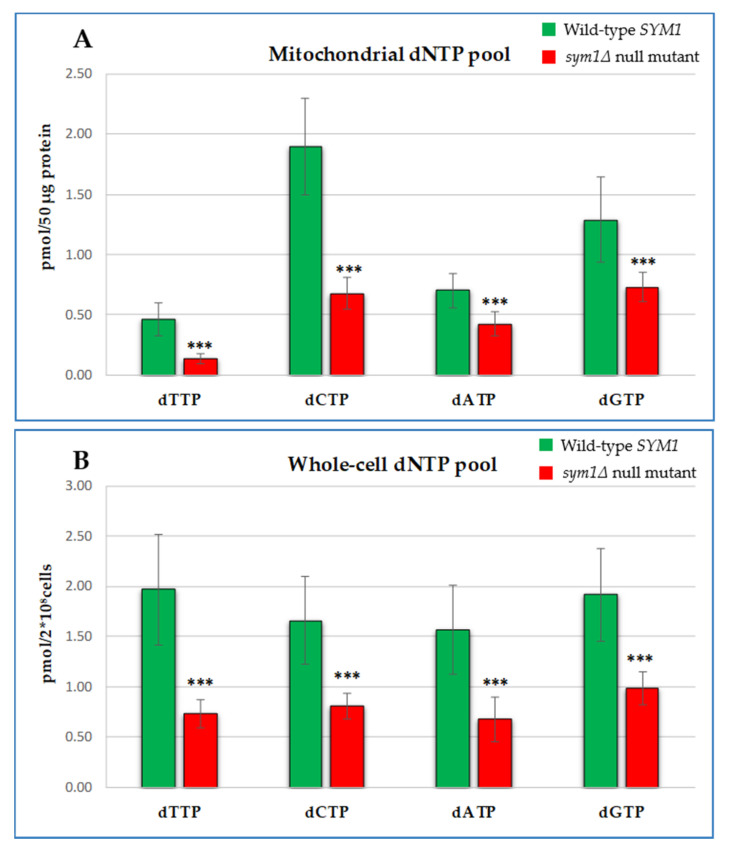
Mitochondrial (**A**) and whole cell (**B**) dNTP amount of the wild-type strain (green bar) and of the null mutant strain (red bar) grown for 24 h at 37 °C in SC medium supplemented with 0.6% glucose and 2% ethanol. Data are represented as the mean of at least eight values ± SD. Mitochondrial dNTP pool was reported as the amount of dNTP (in picomoles) per 50 µg of mitochondrial protein. Whole cell dNTP pools were reported as the amount of dNTP (in picomoles) per 2 × 10⁸ cells. Statistical analyses were performed using a two-tail unpaired Student’s *t* test: *** *p* < 0.001.

As shown in Figure 3A, yeast mitochondria of the *sym1Δ* mutant displayed a significative decrease in the levels of all the dNTPs, with the amount of dTTP and dCTP being down to about 30% of the *SYM1* wild-type content, and those of dGTP and dATP to about 50% of the corresponding values of the wild-type strain. Therefore, it appears that as in the *Mpv17^−/−^* mouse and MPV17 deficient human fibroblasts, the instability of mtDNA in *sym1∆* yeast mitochondria, is associated with a decrease of dNTP levels. This provides strong evidence that the cause of the mtDNA deletion and depletion in Sym1 deficient cells is a shortage of precursors for DNA synthesis. Yeast lacks the deoxyribonucleoside kinase activities of the salvage pathway, making the mitochondrial dNTP pool entirely dependent on the direct transport of dNTPs synthesized de novo in the cytoplasm by ribonucleotide reductase (RNR) activity. Thus, we set out to explore the impact of the *SYM1* deletion on dNTP availability in the whole cell. We observed that the decrease of dNTPs was not limited to only the mitochondrial compartment, but it is comprised of the whole cell dNTP pool (Figure 3B). 

**Figure 4:**
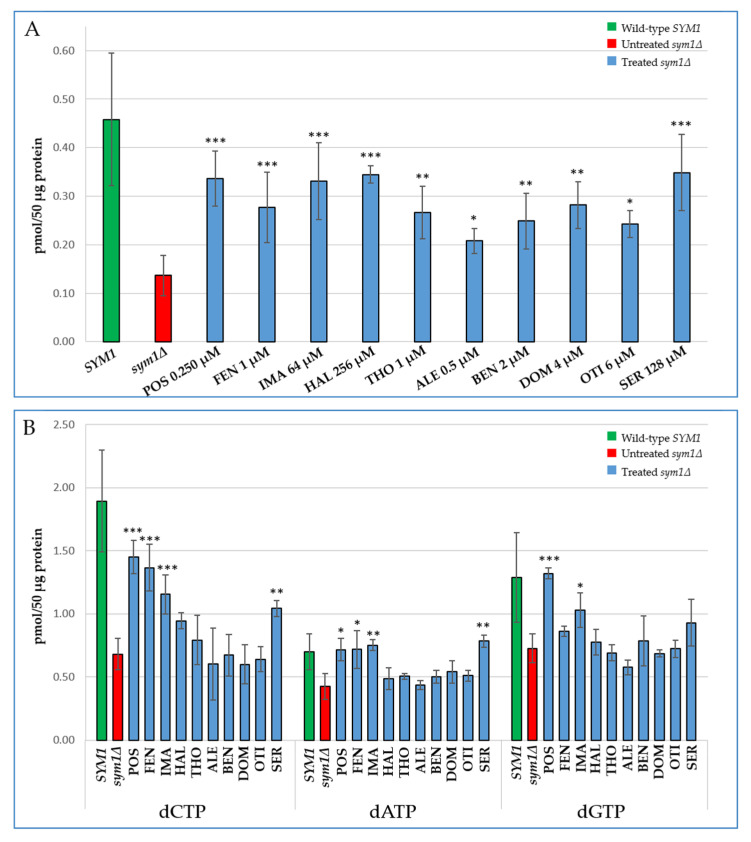
Effect of the identified drugs on mitochondrial dTTP (**A**), dCTP, dATP and dGTP (**B**) amounts of the *sym1∆* null mutant strain. Cells were grown for 24 h at 37 °C in SC medium supplemented with 0.6% glucose and 2% ethanol. The concentration of each drug is specified in panel A. Data are represented as the mean of at least three values ± SD. Statistical analyses were performed using ANOVA followed by a Bonferroni’s post hoc test comparing treated (blue bars) versus the untreated mutant (red bar) in which the compound vehicle DMSO was added: * *p* < 0.05; ** *p* < 0.01; *** *p* < 0.001.

We investigated whether the reduction of the mitochondrial DNA instability in the *sym1Δ* mutant treated with the identified drugs was the result of an increase in the mitochondrial dNTP pool. Thus, we performed mitochondrial dNTP extraction from the *sym1Δ* mutant treated with the beneficial molecules at the concentrations used for mitochondrial DNA instability analysis (Figure 4). 

Addition of all molecules resulted in a significant increase of the mitochondrial dTTP pool in the *sym1∆* strain (Figure 4A). Coherently with the mtDNA instability analysis, Alexidine HCl was the least effective molecule, leading to a moderate increase of dTTP (about 1.5-fold). Treatment with Fenticonazole nitrate, Thonzonium bromide, Benzethonium Chloride, Domiphen bromide and Otilonium bromide doubled the level of dTTP, whereas the addition of Posaconazole, Imazalil, Haloperidol and Sertraline HCl tripled the levels of this nucleotide with respect to the untreated mutant strain. Not all molecules appeared similarly effective in increasing other dNTPs (Figure 4B).

Since we also observed a decrease in the total nucleotide pool, we investigated the effect of molecules in whole cell dNTPs, obtaining results similar to those achieved when the mitochondrial pool was analysed (Supplementary Figure S5).

### 2.4. Characterization of the Effects of the Identified Molecules on other MDS Yeast Models: The rnr2 and mip1 Mutants

In order to potentially extend the use of the identified drugs to a wider range of MDS patients, we analysed the effect of these molecules on mtDNA stability in two other MDS yeast models. One was characterized by a decrease in the dNTP pool due to defective ribonucleotide reductase activity; the other presented mtDNA instability due to defective mitochondrial polymerase activity, that can be corrected by increasing amounts of dNTPs, the substrates of the enzyme. 

**Figure 5:**
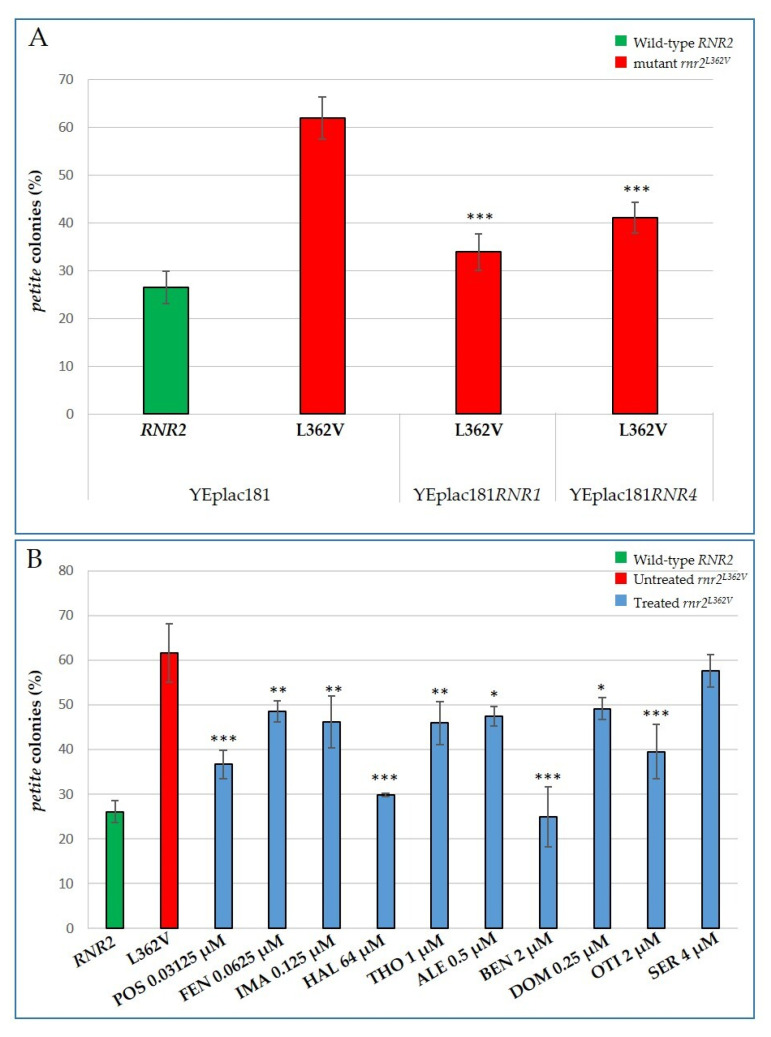
Increased dNTP pool availability reduces the *petite* colonies production of the *rnr2^L362V^* mutant strain. Cells were grown in YP medium supplemented with 2% glucose at 36 °C. More than 4000 colonies/strain were scored. All values are means of three independent experiments ± SD. (**A**) The percentage of *petite* colonies produced in the wild-type *RNR2* and in *rnr2^L362V^* mutant strains combined with *RNR1* or *RNR4* overexpression. Statistical analyses were performed using ANOVA followed by a Bonferroni’s post hoc test comparing the *petite* colonies production of *rnr2^L362V^* transformed with YEplac181*RNR1* or YEplac181*RNR4* versus *rnr2^L362V^* transformed with empty YEplac181: *** *p* < 0.001. (**B**) Beneficial effect of the identified drugs on *petite* colonies production of the *rnr2^L362V^* mutant strain. Statistical analyses were performed using ANOVA followed by a Bonferroni’s post hoc test comparing treated (blue bars) versus the untreated mutant (red bar) in which the compound vehicle DMSO was added: * *p* < 0.05; ** *p* < 0.01; *** *p* < 0.001.

We created the first model by mutating the *RNR2* gene, the ortholog of the human gene RRM2B, which encodes the small p53-inducible ribonucleotide reductase (RNR) subunit (p53R2), the expression of which is essential for DNA repair and mtDNA synthesis in postmitotic cells [36]. Due to the key role of the RNR complex in the biosynthesis of dNTPs, the limited availability of precursors for mtDNA synthesis is the cause of mitochondrial genome deletion and depletion in RRM2B deficiency. Taking advantage of the high degree of conservation between human RRMB2 and its yeast ortholog Rnr2, we introduced several *RNR2* mutations corresponding to human pathological variants, into *S. cerevisiae*. The L362V substitution equivalent to the human L317V clinical variant is the alteration that showed the most pronounced mtDNA instability, increasing the percentage of *petite* colonies by about three-fold compared to the wild-type strain (Figure 5A). This mutation changes Leu362 (hLeu317), which is not only a highly conserved residue between different species, but is also located close to two other residues, Ser361 (hSer316) and Tyr376 (hTyr331). These residues are considered crucial for the interaction between p53R2 and the large subunit R1 and are therefore essential for the proper formation of the RNR complex [37]. 

We first tested whether the increase of the dNTP pool produced by overexpressing the *RNR1* or *RNR4* gene, coding, respectively, the large and small subunit of the RNR complex, a dNTP checkpoint enzyme, could rescue the *petite*-inducing phenotype of the mutant. To do so, we transformed the *rnr2^L362V^* mutant with a multicopy plasmid carrying *RNR1* or *RNR4* and we evaluated the frequency of *petite* colonies. The increased availability of dNTPs appears to prevent mitochondrial genome instability (Figure 5A). We then investigated whether the beneficial molecules capable of increasing the dNTP pool in the *sym1* mutant were also able to reduce the frequency of *petite* colonies in the *rnr2^L362V^* mutant strain (Figure 5B). With the exception of Sertraline HCl, the addition of all molecules significantly improved the mtDNA stability of the *rnr2^L362V^* mutant cells, although with different intensities. Haloperidol and especially Benzethonium chloride showed a more marked effect. The percentage of *petite* colonies in the mutant treated with Haloperidol was halved compared to the untreated mutant, and was reduced by 70% in the mutant treated with Benzethonium chloride, bringing the frequency of *petite* colonies to lower levels than the wild-type (Figure 5B). 

**Figure 6:**
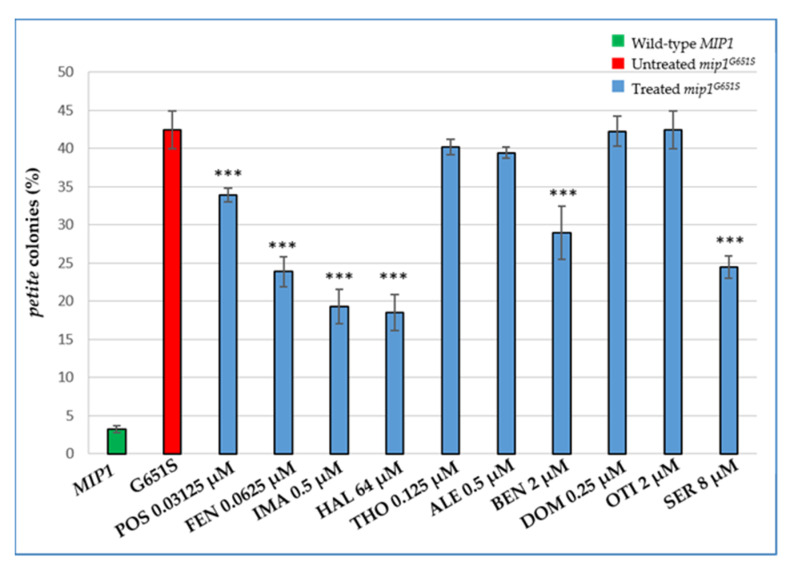
Beneficial effect of the identified drugs on *petite* colonies production of the *mip1^G651S^* mutant strain. Cells were grown in YP medium supplemented with 2% glucose at 28 °C. More than 4000 colonies/strain were scored. All values are means of three independent experiments ± SD. Statistical analyses were performed using ANOVA followed by a Bonferroni’s post hoc test comparing treated (blue bars) versus the untreated mutant (red bar) in which the compound vehicle DMSO was added: *** *p* < 0.001.

The second yeast model tested depends on *MIP1,* an ortholog of the human gene POLG, encoding the catalytic subunit of human mtDNA polymerase γ. We exploited the yeast strain harbouring the mutant allele *mip1^G651S^* equivalent to the human POLG G848S clinical variant, one of the most common POLG mutations [38] and associated with a 1000-fold decrease in the in vitro catalytic efficiency [39]. It has been previously demonstrated that the mtDNA instability of this strain is drastically reduced when the dNTP pool increases following the deletion of *SML1*, an RNR inhibitor [40]. We therefore investigated whether the beneficial molecules identified in MPV17-related MDS, capable of increasing dNTPs levels in the *sym1∆* strain, were also able to reduce the mtDNA instability of the *mip1^G651S^* mutant strain. The addition of Thonzonium bromide, Alexidine HCl, Domiphen bromide or Otilonium bromide did not result in a decrease of *petite* colony mutability. Posaconazole and Benzethonium chloride treatment led to a decrease in production of *petite* colonies between 20% and 30%, whereas the addition of Fenticonazole nitrate, Imazalil, Haloperidol and Sertraline HCl, as the most effective compounds, resulted in a decrease in *petite* colonies generation of between 45% and about 55% (Figure 6).

The effects of the molecules on the mtDNA instability of *sym1^R51W^*, *rnr2^L362V^* and *mip1^G651S^* mutants are summarized in Table 2. 

## 3. Discussion

Mitochondrial DNA depletion syndromes (MDS) are often caused by defective proteins involved in the mtDNA replication machinery, such as POLG, or in dNTP metabolism, such as p53R2 [1]. In addition to mutations in these genes, for which the mechanism linking mutations and mtDNA depletion has been clarified, mutations in the MPV17 gene were also described as a cause of an hepatocerebral form of MDS. Discovered about 30 years ago, MPV17 encodes a protein embedded in the IMM, but its role in mtDNA maintenance remains an open question. So far, the leading hypothesis is that MPV17 deficiency might affect mitochondrial dNTP metabolism, leading to an insufficient availability of dNTPs and consequent mitochondrial DNA depletion. For this reason, MPV17-related disease was classified to the group of mtDNA disorders caused by mitochondrial dNTP perturbation [12,14]. More specifically, several findings point to perturbed guanosine metabolism as critical to the MPV17-related disorder. Indeed, it was shown that: (i) MPV17 deficiency in zebrafish results in a strong reduction of pigment cell iridophores, mainly constituted by guanine [13]; (ii) the loss of function of deoxyguanosine kinase (DGOUK) and MPV17 are associated with similar hepatocerebral syndromes [4,41]; (iii) MPV17 KO mice showed an elevated ribonucleoside guanosine monophosphate (rGMP) level in mtDNA [42]. However, the significant impairment of dihydroorotate dehydrogenase (DHODH) activity in *mpv17^−/−^* null zebrafish mutants, coupled with the rescue of their phenotype after supplementation with pyrimidine precursors, link the loss of MPV17 to pyrimidine de novo synthesis [14]. 

Here we showed that, in *S. cerevisiae,* the deletion of the MPV17-ortholog, *SYM1*, resulted in a general decrease in all four mitochondrial nucleotides, and demonstrated that the nucleotide reduction is confined to the entire cell compartment. A decrease in the whole cell dNTP pool could be caused by an aberrant production of ATP due to an OXPHOS impairment; however, the observed decrease in *sym1Δ* yeast cells occurs independently from mtDNA instability [7] and the lower levels of dNTPs could be a consequence of the defective control of TCA cycle intermediates, as previously reported [7]. Indeed, a reduction or an imbalance in TCA cycle intermediates could impair cytosolic nucleotide biosynthesis. As *S. cerevisiae* lacks a deoxynucleotide salvage pathway, the mitochondrial dNTP pool is exclusively dependent on cytosolic dNTP transport; as such, the reduction of the cytosolic dNTP pool could be reflected by a decrease of mitochondrial nucleotides.

To date, the treatment of MDS has focused on symptomatic management which provides relief but does not stop the progression of the disease [18]. However, in recent decades, thanks to the advances in understanding the molecular basis of several MDSs, new “precision medicine” strategies have emerged, often with promising preclinical and clinical results [16]. Among these approaches, supplementation of deoxyribonucleosides (dNs) has been proposed as a powerful strategy for the treatment of MDSs caused either by defective nucleotide metabolism (such as RRM2B and MPV17-related diseases) or an impairment of the mtDNA synthesis machinery (such as POLG-related diseases) [12,43,44,45]. 

The investigation of putative therapeutic strategies for mitochondrial diseases can greatly benefit from the use of a simple, fast growing and low-cost organism like *S. cerevisiae*. Thanks to the development of the “drug drop test”, it is now possible to screen a large number of molecules in a short time for their potential relevance in a therapeutic context. In this report we exploited this method and identified ten pharmacological compounds able to rescue the defective yeast phenotype caused by the *sym1^R51W^* mutation. We also demonstrated that all these molecules were able to determine a significant increase in the dTTP pool and strongly improve the mtDNA stability of *sym1∆* mutant cells. These results evidence that a decreased availability of DNA building blocks is the cause of the mtDNA maintenance defect in Sym1 deficiency. Not all molecules were able to increase other dNTPs, but these discrepancies could be due to a lower sensitivity of the assay in detecting small changes of these dNTPs (as possibly indicated by the calibration curves; Supplementary Figure S6). However, we cannot exclude that dTTP, the less abundant among the four dNTPs in yeast and mammalian mitochondria [46], could be the limiting factor, and that therefore the rescue of dTTP, more than that of other dNTPs, may have beneficial effects. 

Posaconazole, Fenticonazole nitrate and Imazalil belong to the class of azoles with antifungal activity [47]. These drugs inhibit the CYP51 enzyme (lanosterol 14-α-demethylase) encoded by the *ERG11* gene, a key enzyme involved in the biosynthesis of ergosterol, the major sterol of the fungal IMM [48]. Sterols are the most important hydrophobic lipids of eukaryotic cell membranes, playing a central role in many biological processes, such as endocytosis [49] and stabilization of membrane proteins [50] and in maintaining proper membrane permeability and fluidity [51]. Interestingly, Haloperidol, used in human therapy as an antipsychotic medication owing to its strong antagonism of the D2 dopamine receptor [52], also acts on the ergosterol pathway in yeast. It inhibits both C8-C7 sterol isomerase (encoded by *ERG2* gene) and C-14 reductase (encoded by the *ERG24* gene), thus reducing ergosterol content [53,54]. Given its important role as a modulator of membrane properties, a severe reduction in its levels, due to the deletion of *ERG* genes or the presence of inhibitors of sterol biosynthesis (e.g., azoles), is deleterious for the yeast cell [55,56]. However, these drugs were able to rescue the mtDNA instability caused by mutations in *SYM1*, *MIP1* or *RNR2* genes, suggesting that modulation of ergosterol levels can have a beneficial effect on increasing the permeability of mitochondrial membranes.

In mammalian cells, cholesterol performs many of the functions that ergosterol performs in fungal cell membranes [57]. It is a modulator of the bilayer structure of membranes and affects several membrane proteins such as ion channels and transporters of several metabolites, such as adenine nucleotides [58], 2-oxoglutarate, glutathione [59], citrate [60], phosphate [61] and pyruvate [62]. Therefore, in human cells characterized by MPV17 deficiency, a moderate reduction of cholesterol could also have beneficial effects. Currently, statins are used as cholesterol-lowering drugs for cholesterol-related diseases. However due to side effects, their use is limited for the treatment of MPV17-related diseases that require long-term administration. Indeed, these drugs, by inhibiting HMG-CoA reductase, the rate-limiting enzyme of the mevalonate pathway, prevent the biosynthesis of other biologically important substances derived from mevalonate. The human CYP51A1, an ortholog of yeast *CYP51*, could be a more specific molecular drug target because its moderate inhibition could specifically reduce cholesterol synthesis without affecting other collateral pathways such as the mevalonate pathway. Despite a high degree of conservation between fungal and human enzymes, many of the CYP51 inhibitors have exhibited a low affinity for CYP51A1 and, in fact, are used as antifungal agents in human therapy. For an effective treatment of the disease associated with mutations in MPV17, specific inhibitors of the activity of CYP51A1 should therefore be identified. Recently, through comparative structural and functional studies of CYP51 orthologs from different biological kingdoms, it was possible to identify the structural features that make the human enzyme resistant to inhibition. Based on these observations, potent stoichiometric human CYP51 inhibitors were synthesized [63,64]. 

Regarding the other molecules identified in this study, there is little or no information about their mechanism of action in yeast, therefore the cause of the beneficial effects remains widely speculative. Antifungal activity, due to inhibition of the vacuolar V-ATPase proton pump, have been reported for Alexidine HCl and Thonzonium bromide [65,66,67]. Interestingly impairment of V-ATPase leads to phenotypic defects similar to those observed in *ERG2* mutants [68]. Based on these observations, it is reasonable to speculate that these two V-ATPase inhibitors could induce an alteration of the content of ergosterol in the IMM, thus enabling flows in *sym1* mutant cells. An alteration of the flows also appears the most probable mechanism of action for Benzethonium chloride and Sertraline HCl. In fact, the former induces membrane perturbation in yeast, probably by intercalating into the lipid bilayer [69,70], whereas the latter interacts with phospholipid membranes [71], inducing membrane ultrastructural changes in yeast. 

Most of the identified beneficial molecules (except Sertraline HCl) were also able to alleviate the mtDNA instability of *rnr2* mutant cells, whereas fewer molecules were effective in the *mip1* mutant strain. The different responses of *rnr2* and *mip1* mutants to treatment may be due to different pathomechanisms of *RNR2- or MIP1*-related mtDNA instability. In the *rnr2* mutant strain, the increased mitochondrial genome instability is due to a limited availability of dNTPs, as in *sym1∆* cells. In Mip1-deficient cells, mtDNA instability is the result of defective DNA polymerase activity. Replenishment of the missing or insufficient nucleotides in the *rnr2* mutant strain, following supplementation with the drugs, may be sufficient to counteract the instability of mtDNA. Conversely, the increase in the dNTP pool obtained by adding Thonzonium bromide, Alexidine HCl, Domiphen bromide or Otilonium bromide in the *mip1* mutant strain, may not be sufficient to bypass the catalytic defect of the enzyme and therefore may not improve the stability of the mitochondrial genome. However, the other six tested molecules were able to significantly reduce the mtDNA instability of the *mip1^G651S^*mutant strain. This feature would be very interesting since POLG mutations are associated with 10–25% of progressive external ophthalmoplegia (PEO) and >10% of ataxia cases and are the most common cause of mitochondrial epilepsy [38]. 

All the molecules, but Imazalil, identified in this study are FDA-approved and so their declared safety makes them potentially easy to be repurposed, even if some of these are prescribed for external use only. So far, these drugs are used for the treatment of various diseases including mycoses, bacterial infections, psychotic disorders, depression and irritable bowel syndrome. For all these molecules, the target in mammals is known, although the mechanism of action is not clear for all of them. Some of the targets identified in humans do not exist in yeast, thus suggesting that the biological activity of these drugs in yeast is probably due to an action on one or more secondary targets.

Further investigations are required to decipher the specific molecular mechanism of action of the identified drugs and it will be useful to demonstrate the beneficial effect of these drugs at a multiorgan level. Nevertheless, the identification of molecules capable of reducing the mtDNA instability in different MDS yeast models opens new and “low-cost” opportunities and serves as an initial proof-of-concept to develop drugs for MDS. 

## 4. Materials and Methods

### 4.1. Yeast Strains and Culture Media

Yeast strains used in this study were: (i) BY4741 *sym1::kanMX4* (*MATa; his3Δ1 leu2Δ0 met15Δ0 ura3Δ0 sym1::kanMX4*) transformed with the empty centromeric vector pFL38 or the vector carrying the *SYM1* wild-type allele or the mutant *sym1^R51W^* allele, as previously described [7,34]; (ii) BY4741 (*MATa; his3Δ1 leu2Δ0 met15Δ0 ura3Δ0*); (iii) BY4741 *rho^0^*, obtained by treating the BY4741 wild-type strain with 20 µg/mL ethidium bromide and monitoring the lack of mtDNA by 4′,6-diamidino-2-phenylindole (DAPI) staining; (iv) DWM-5A *mip1::KanR* (*Mata ade2-1 leu2-3,112 ura3-1 trp1-1 his3-11,15 can1-100 mip1::KanR*) transformed with the vector pFL39 carrying the *MIP1* wild-type or the mutant *mip1^G651S^* mutant allele, as previously reported [40] and (v) W303-1A (*MATa ade2-1 his3-11,15 leu2-3,112 trp1-1ura3-1*) [72]. *RNR2* was amplified with the oligonucleotides reported in Supplementary Table S1 and *Pst*I-cloned, under its natural promoter, in the centromeric plasmids pFL38 and pFL39 [73]. Since *RNR2* deletion makes the strain inviable, strain W303-1A was transformed with pFL38*RNR2* using the lithium acetate, single-stranded DNA, polyethylene glycol method [74]. Then, the genomic copy of *RNR2* was disrupted through one-step gene disruption [75], using the KanMX4 cassette amplified from pFA6-KanMX4 [76] and oligonucleotides containing the sequence flanking the *RNR2* open reading frame (ORF), and selecting the disrupted colonies on YP medium supplemented with 2% glucose and 200 mg/mL G418 sulfate (Formedium™, Hunstanton, UK). The mutation L362V was introduced in pFL39 through mutagenic overlap PCR [77], thus obtaining the plasmid pFL39*rnr2^L362V^*. The plasmids were introduced in the W303-1A*rnr2∆*/pFL38*RNR2* strain, and the wild-type (wt) copy of *RNR2* on pFL38 was removed through plasmid shuffling on SC medium supplemented with 2% glucose and 1 g/L 5-FOA (Formedium™, Hunstanton, UK) as previously reported [78], thus obtaining the W303-1A*rnr2Δ/*pFL39*rnr2^L362V^* strain. Mutant *RNR2* strains overexpressing *RNR1* or *RNR4* were obtained by transforming the W303-1A*rnr2Δ/*pFL39*rnr2^L362V^* strain with the empty episomal vector YEplac181 and with the vector-borne *RNR1* or RNR4, cloned as previously reported [79].

Cells were cultured in SC medium (0.69% yeast nitrogen base without amino acids, Formedium™, Hunstanton, UK), supplemented with 1 g/L dropout mix [78] without uracil or tryptophan (to keep the pFL38 or pFL39 plasmid, respectively) or in YP (0.5% yeast extract, 1% peptone, Formedium™, Hunstanton, UK). Media were supplemented with various carbon sources at various concentrations as indicated in liquid phase or after solidification with 20 g/L agar (Formedium™).

### 4.2. High-Throughput Screening: Drug Drop Test

The primary screening was performed with the Selleck Chemicals drug library (Selleck Chemicals, Houston, TX, USA), which contains 1018 compounds, and with 6 molecules of the Tebu-Bio (Tebu-Bio, Yvelines, France) or the FDA-approved Prestwick chemical libraries (Prestwick Chemical, Illkirch-Graffenstaden, France). The screening was performed as reported by Couplan et al. [24]. Specifically, wild-type (*SYM1*) and mutant strains (*sym1^R51W^* and *sym1Δ*) were inoculated in YP medium supplemented with 2% ethanol and incubated at 28 °C under constant shaking. After 3 d of growth, cells were inoculated in YP medium supplemented with 2% glucose, grown for 6 h at 28 °C and then 1.5 × 10^5^ (*sym1^R51W^*) or 2 × 10^5^ (*sym1Δ*) cells were spread on 12 × 12 square plates containing 90 mL of YP solid medium supplemented with 4% ethanol and 50 µg/mL streptomycin and 50 U/mL penicillin. After the seed, 31 sterile filters of 6 mm of diameter were put on the agar surface and soaked with 2.5 µL of each compound at a concentration of 10 mM dissolved in DMSO, except for one disk which was spotted with 2.5 μL of DMSO as a negative control. As a positive growth control, the wild-type strain (*SYM1*) was used for spotting an appropriate number of cells to obtain the same density (cell/cm^2^) as the plated mutant cells. Positive hits were identified after 72–96 h incubation at 37 °C by the halo of growth around the filters. The secondary screening of positive compounds selected by the primary screening was performed under the same conditions, except that a lower number of disks were added on each plate, among which, one was spotted with DMSO.

### 4.3. Petite Frequency and Fitness Test

For *petite* frequency measurement, cells were pre-grown at 28 °C in SC medium supplemented with 2% ethanol to counterselect the *petite* cells already present in the population. Then the conditions differed depending on the strain analyzed. For *SYM1*, cells were inoculated in SC liquid medium supplemented with 2% glucose and incubated for 4 h at 28 °C. Then 2% ethanol was added in the medium together with drugs or DMSO and the cultures were shifted to 37 °C. For *RNR2* and *MIP1*, cells were inoculated in YP liquid medium supplemented with 2% glucose in the presence of the molecules or DMSO. After 15 generations of growth at 37 °C (*SYM*1), 36 °C (*RNR2*) and 28 °C (*MIP1*), cells were plated on SC agar plates supplemented with 2% ethanol and 0.4% glucose at a dilution giving approximately 200 cells/plate. *Petite* frequencies were defined as the percentage of colonies showing the *petite* phenotype after 5 d of incubation at 28 °C [78]. To exclude that the reduction of *petite* colonies observed was due to a negative effect on the division time or on the viability of *petite* mutants, the effect of the drugs on the *petite* fitness was analyzed by growing an equal number of isogenic *rho^+^* and *rho^0^* BY4741 cells in the same culture in the presence of the tested molecules or DMSO. Cells were plated after 15 generations on SC agar plates supplemented with 2% ethanol and 0.4% glucose at a dilution giving approximately 200–250 cells/plate to determine the ratio of respiratory sufficient and respiratory deficient (*petite*) colonies.

### 4.4. Whole Cell Dexoxyribonucleoside Triphosphate Extraction

Wild-type SYM1 and null-mutant *sym1Δ* cells were pregrown at 28 °C in SC supplemented with 2% ethanol to counterselect the *petite* cells that could be present in the population. Subsequently, cells were exponentially grown in SC supplemented with 2% glucose and transferred to SC medium supplemented with 0.6% glucose and 2% ethanol and incubated for 24 h at 37 °C. In the latter medium, glucose is the preferential carbon source, whereas ethanol acts as a stress factor for the *sym1∆* strain, as previously reported [7]. A total of 2 × 10^8^ cells were harvested by centrifugation (at 6000 rpm for 5 min) and nucleotide extracts were prepared by methanol extraction and boiling according to a previously reported method [80]. The harvested cells were resuspended in 1 mL of 60% ice-cold methanol and incubated for 2 h at −20 °C. They were subsequently heated for 3 min at 99 °C to destroy residual enzymatic activity of nucleases and nucleotide kinases which could interfere with the enzymatic dNTP assay, and then centrifuged at 17,000× *g* for 15 min. Extracts were dried using a SpeedVac vacuum concentrator and the residues were resuspended in 50 μL of ice-cold deionized water and later used for quantification of the four dNTPs by the DNA polymerase assay (see below).

### 4.5. Mitochondrial Extraction and Separation of Mitochondrial and Cytosolic dNTPs

Yeast cells were grown as described in the previous paragraph and 2 × 10^8^ cells were harvested by centrifugation (at 6000 rpm for 5 min). After two deionized water washes, each pellet was resuspended in 400 μL of 0.6 M sorbitol and 5 mM dithiothreitol (DTT, AppliChem GmbH, Darmstadt, Germany), then incubated for 20 min at room temperature. Samples were centrifuged at 7000 rpm for 30 s and the pellet was resuspended in 400 μL of 1.2 M sorbitol, 10 mM Tris-HCl pH 7.5 and zymolyase 20T (12 mg/mL; Nacalai Tesque, Inc., Kyoto, Japan); enzymatic digestion of the cell wall was monitored using a Cary spectrophotometer at 800 nm and it was stopped when an 80–90% decrease in the optical density was observed. To separate the cytosolic nucleotides from those from mitochondria, differential centrifugation was performed as described by Pontarin et al. [81]. In particular, spheroplasts formed after enzymatic digestion were centrifuged for 25 min at 17,000× *g* at 4 °C to separate nuclei and mitochondria from the cytosol. The obtained pellet (nuclei and mitochondria) was washed with 0.5 mL of extraction buffer (0.6 M sorbitol, 20 mM Tris-HCl pH 7.5, 1 mM EDTA) and then centrifuged as above. This washing step is necessary to remove any remaining cytosolic dNTPs from the nuclei. The pellet, now containing the mitochondrial dNTP pool, was resuspended in 50 μL of extraction buffer, followed by quantification of the protein concentration by Bradford’s method [82]. A 50 μg aliquot of protein from each mitochondrial preparation was used to extract mitochondrial dNTPs. Subsequently, a centrifugation for 25 min at 17,000× *g* at 4 °C was performed, and the resulting pellet was resuspended in 1 mL of ice-cold 60% methanol and incubated for 2 h at −20 °C. The remaining steps were the same as those used for the whole cell dNTP pool except for the volume of ice-cold deionized water in which extracts were resuspended, which was 10 μL.

### 4.6. DNA Polymerase Assay

dNTP concentrations were determined using a DNA polymerase assay [83]. Two primers (p13-mer and p27-mer) labelled at the 5′-end with DY682 fluorophore, annealed to their complementary oligonucleotides, were used and the elongation reaction was catalyzed by the Klenow fragment of *E. coli* DNA polymerase I (DNA Polymerase I, Large (Klenow) Fragment, New England BioLabs). Single strand templates were mixed with primers in a 2:1 molar ratio in order to obtain double-stranded oligonucleotides (dimers). Reactions were performed in 80 mM Tris-HCl pH 7.8 and 40 mM NaCl, heated for 5 min at 85 °C and then allowed to slowly cool down to 26 °C [83]. The enzymatic assay was carried out using a 4:1 molar ratio of unlabeled dimer to labeled dimer. The reaction conditions for pyrimidine triphosphate determination were 4.5 pmol of p13/tT, 4.5 pmol of p27/tC, 100 pmol of dATP in 10 mM Tris-HCl pH 7.9, 10 mM MgCl_2_, 50 mM NaCl, 1 mM DTT. The reaction conditions for purine triphosphate determination were 4.5 pmol of p13/tA, 4.5 pmol of p27/tG, 100 pmol of dTTP in 10 mM Tris-HCl pH 7.9, 10 mM MgCl_2_, 50 mM NaCl, 1 mM DTT. The final volume was 30 µL including 5 µL of whole cell extract or 10 µL of mitochondrial extract. Reactions were carried out for 20 min at 26 °C, started by the addition of 0.6 U KF and stopped by the addition of an equal volume of stop dye solution (80% formamide, 5 mM EDTA). The products were separated by PAGE electrophoresis on a 12.5% polyacrylamide–urea gel. The gel was subsequently visualized on a ChemiDoc MP Imaging System (Bio-Rad, Hercules, CA, USA) in the far-red spectrum/DyLight 680 setting (excitation: 650–675 nm, emission: 700–730 nm). The amount of fluorescence of each band was later quantified with Image Lab Software (Bio-Rad, Hercules, CA, USA). The quantity of dNTPs in each sample was obtained by interpolation from the calibration curves previously set up with a standard quantity of dNTPs.

## Figures and Tables

**Table 1:** Yeast and mammalian target of the active compounds identified by drug drop test. hERG = ether-a-go-go-related gene; VD = voltage-dependent; NK = natural killer; 5-HT = 5-hydroxytryptamine.

Drug	Target in Yeast	Target in Mammals
POS	Lanosterol 14-α-demethylase (ergosterol pathway)	Lanosterol 14-α-demethylase (cholesterol pathway)
FEN	Lanosterol 14-α-demethylase (ergosterol pathway)	Lanosterol 14-α-demethylase (cholesterol pathway)
IMA	Lanosterol 14-α-demethylase (ergosterol pathway)	Lanosterol 14-α-demethylase (cholesterol pathway)
ITR	Lanosterol 14-α-demethylase (ergosterol pathway)	Lanosterol 14-α-demethylase (cholesterol pathway)
SEC	Lanosterol 14-α-demethylase (ergosterol pathway)	Lanosterol 14-α-demethylase (cholesterol pathway)
HAL	Sterol C8-C7 isomerase (ergosterol pathway)	Dopamine receptors; sigma-1 receptor; 3β-hydroxysterol-∆^8^, ∆^7^ isomerase (cholesterol pathway)
THO	Vacuolar ATPase proton transporter	Vacuolar ATPase proton transporter
ALE	Vacuolar ATPase proton transporter	Vacuolar ATPase proton transporter
BEN	unknown	hERG K-channel
DOM	unknown	hERG K-channel
OTI	unknown	Muscarinic receptor, VD Ca-channel, NK receptor
SER	Phospholipid membranes	Serotonin 5-HT transporter

**Table 2:** Effects of the molecules on the mtDNA instability of *sym1^R51W^*, *rnr2^L362V^* and *mip1^G651S^* mutant strains measured as a reduction of *petite* frequency. +++ strong effect (greater than 50% reduction); ++ medium effect (reduction between 50% and 25%); + mild effect (less than 25% reduction). The evaluation of the molecule’s efficacy was made on the basis of the results obtained at the most effective concentration identified for each mutant strain. n.e. = no effect.

Rescue on mtDNA Instability			
Drug	*sym1^R51W^*	*rnr2^L362V^*	*mip1^G651S^*
POS	+++	++	+
FEN	++	+	++
IMA	+++	++	+++
HAL	++	+++	+++
THO	+	++	n.e.
ALE	+	+	n.e
BEN	+++	+++	++
DOM	++	+	n.e.
OTI	++	++	n.e
SER	++	n.e	++

## Data Availability

Data sharing not applicable.

## Supplementary Materials

The following are available online at https://www.mdpi.com/article/10.3390/ijms222212223/s1.
