# Supplementary material for: A Yeast-Based Repurposing Approach for the Treatment of Mitochondrial DNA Depletion Syndromes Led to the Identification of Molecules Able to Modulate the dNTP Pool
Source: Int J Mol Sci. 2021 Nov 12;22(22):12223. doi: 10.3390/ijms222212223 (PMC8621932; doi:10.3390/ijms222212223)
Supplement: Supplementary file 1 [file ijms-22-12223-s001.zip › ijms-1426366-supplementary.pdf]

Supplementary Figure S1

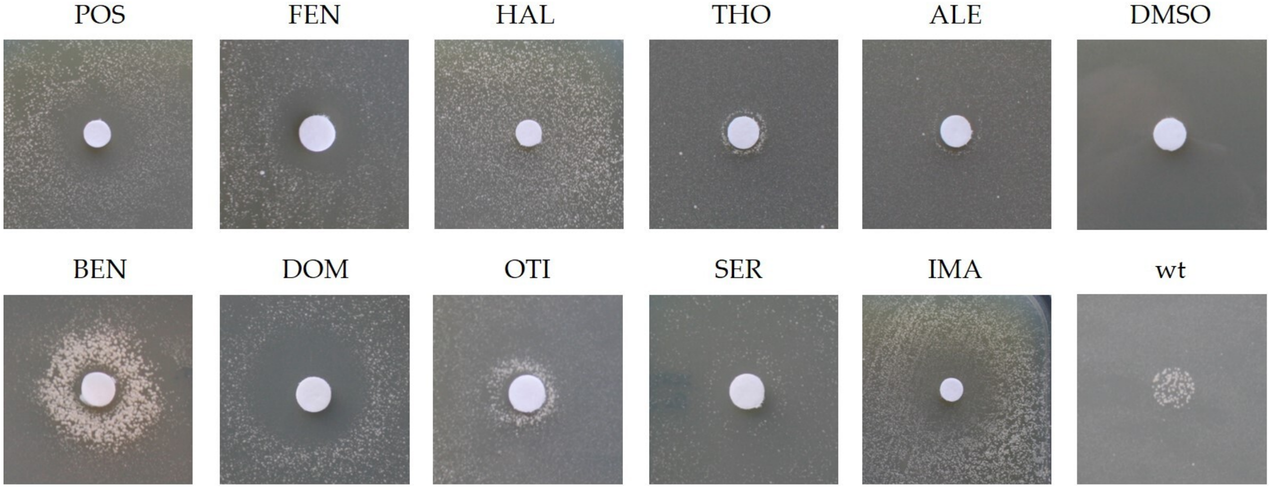

Figure S1. Rescue of the defective OXPHOS growth phenotype of the null mutant *sym1Δ* induced by active compounds. One filter was loaded with the same amount of (DMSO), the solvent in which the molecules were solubilized. As a positive growth control, the wild-type strain was used as described in the Materials and Methods.

Supplementary Figure S2

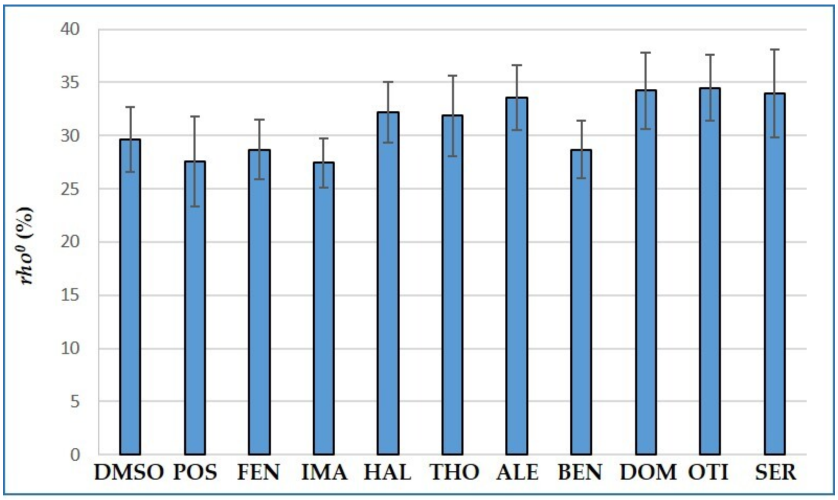

Figure S2. *Rho*^{0} percentage obtained by fitness test performed growing together BY4741 *rho*$^{+}$ and BY4741 *rho*^{0} cells mixed in an equal amount in presence of the active compounds or DMSO. Values are means of three independent experiments ± SD.

Supplementary Figure S3

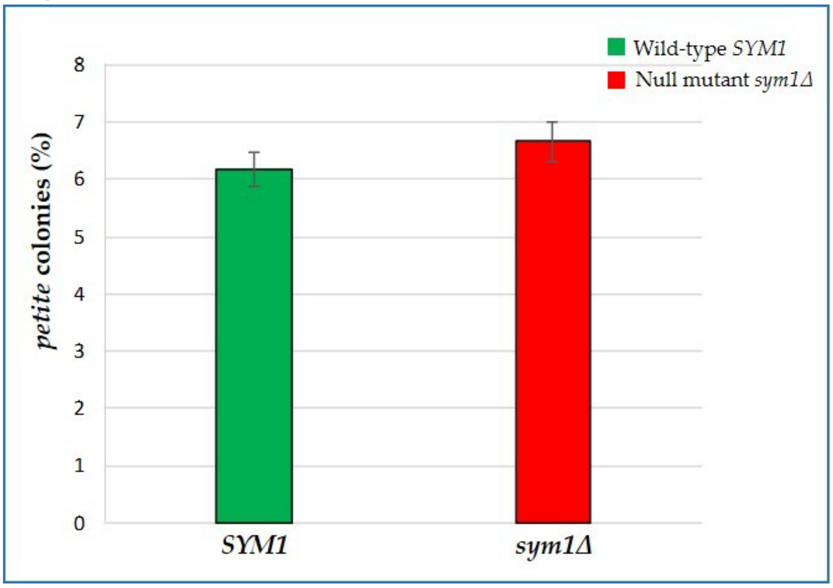

Figure S3. *Petite* frequency of the *SYM1* (green bar) and *sym1Δ* (red bar) strains grown for 24 h at 37 °C in SC medium supplemented with 0.6% glucose and 2% ethanol. More than 4,000 colonies/strain were scored. Values are means of two independent experiments ± SD.

Supplementary Figure S4

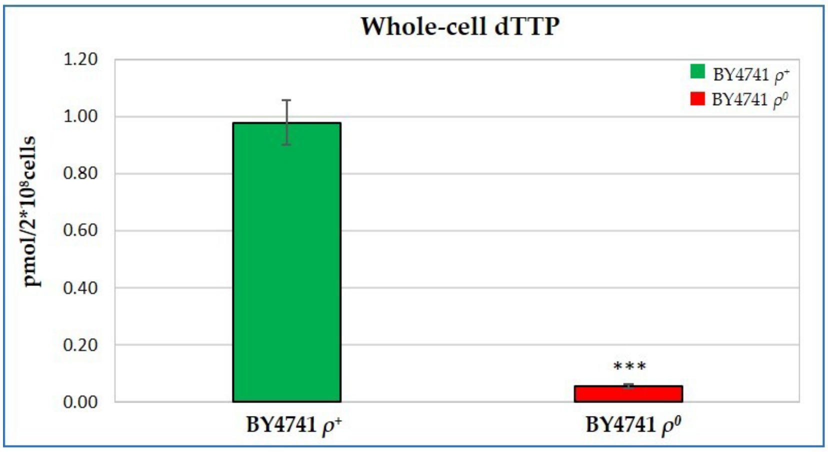

Figure S4. Whole-cell dTTP pool of BY4741 *rho*$^{+}$ (ρ^{+}) and BY4741 *rho* ^{0} (ρ^{0}) grown for 24 h at 37 °C in SC medium supplemented with 0.6% glucose and 2% ethanol. Values are means of three independent experiments ± SD. Statistical analyses were performed using a two-tail unpaired Student's t test comparing BY4741 *rho*^{0} (red bar) versus BY4741 *rho*$^{+}$ (green bar): *** $p < 0.001$.

Supplementary Figure S5

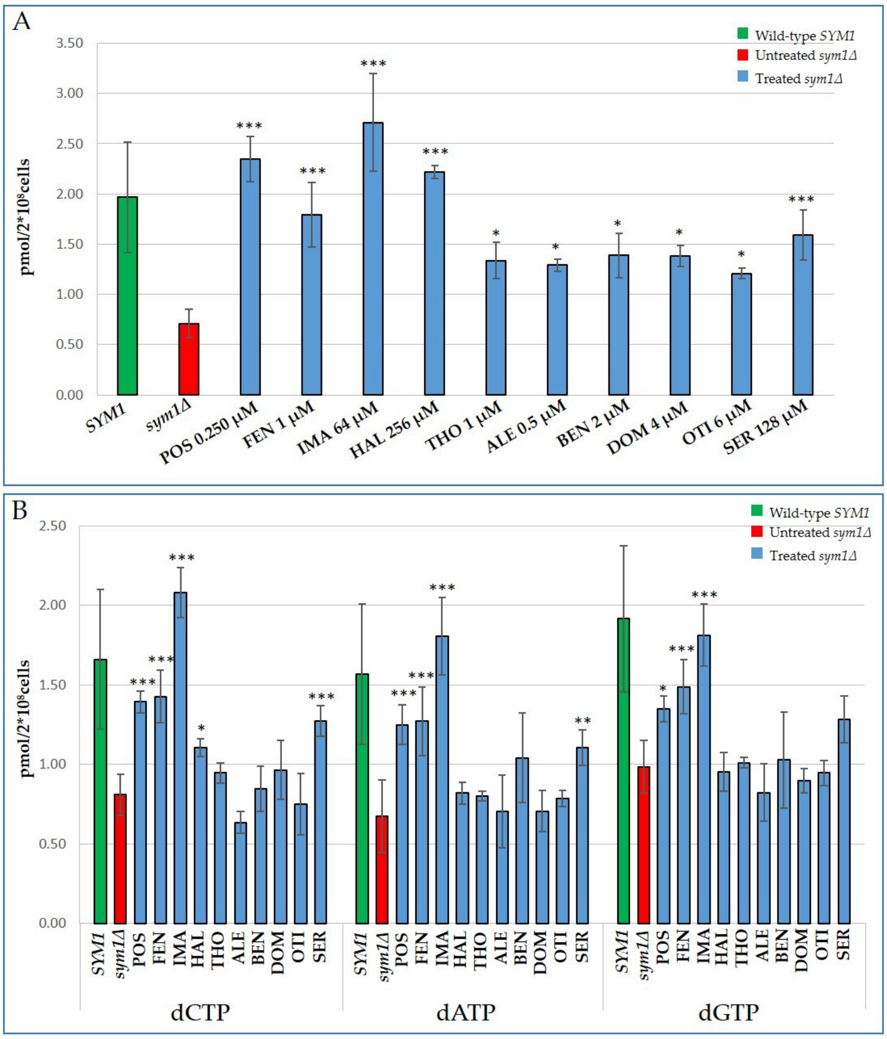

Figure S5. Effect of the identified drugs on whole-cell dTTP **(A)**, dCTP, dATP and dGTP **(B)** amount of *sym1Δ* null mutant strain. Cells were grown for 24 h at 37 °C in SC medium supplemented with 0.6% glucose and 2% ethanol. The concentrations of each drug is specified in panel A. Data are represented as the mean of at least three values ± SD. Statistical analyses was performed using ANOVA followed by a Bonferroni's post hoc test comparing treated (blue bars) versus untreated mutant (red bar) in which the compound vehicle DMSO was added: * $p < 0.05$; ** $p < 0.01$; *** $p < 0.001$.

Supplementary Figure S6

A

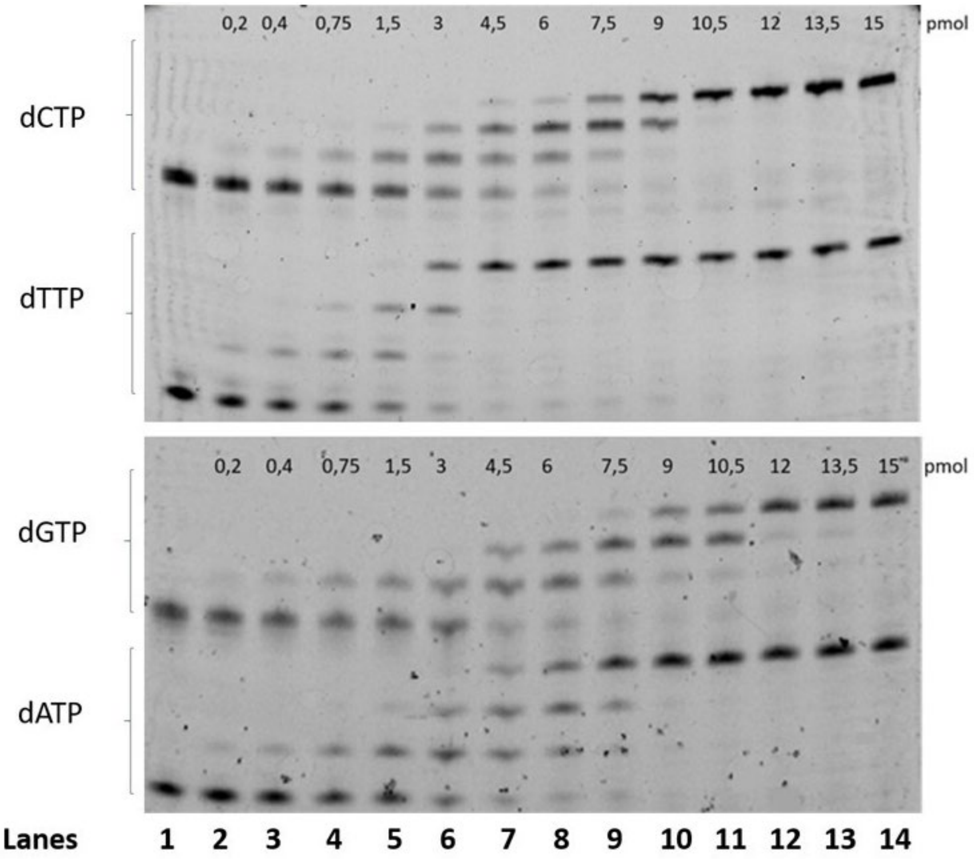

B

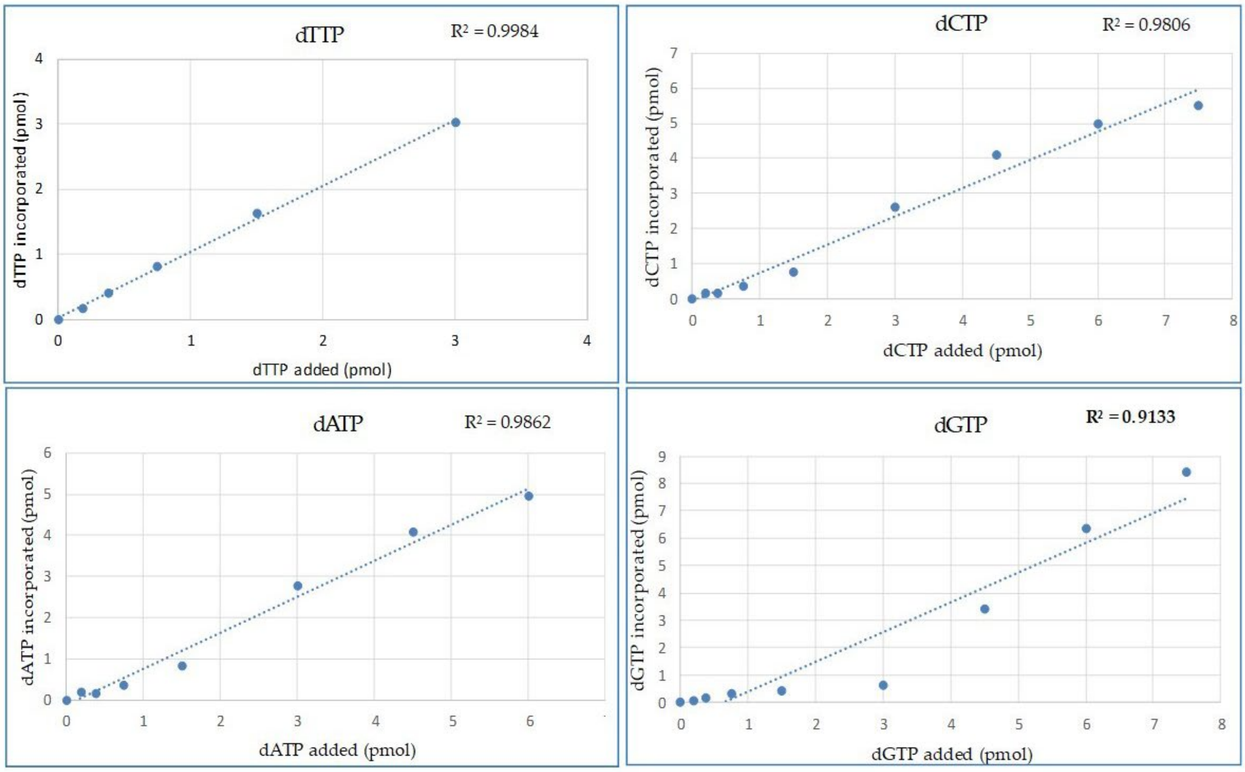

Figure S6. **(A)** Visualization of a urea-PAGE gel of elongated products obtained with the indicated amount of dNTPs. The non-elongated products displayed in lane 1 of both gels were detected when the dimers for the quantification of the two pyrimidines (p13/tT and p27/tC) and those for the quantification of the two purines (p13/tA and p27/tG) were incubated with an excess of dATP and dTTP, respectively, but without the nucleotide to be quantified, thus the p/t dimers remained of its original length. These non-elongated products were used as markers in the gels. Depending on the quantities of dNTPs, primers can be elongated to one, two or three positions leading to products of different lengths corresponding, respectively, to the second, third or fourth band starting from the bottom of the gel. **(B)** Calibration curves for pyrimidines (in the upper part) and for purines (in the lower part). Data are represented as mean values of two independent experiments. For each curve the related R^2 is reported.

Supplementary Table S1

Oligo name	Sequence	Use
*RNR2*DFw	GAATCCAAACTTAATACACGTATTTATTTGTCCAAT TACCGCATAGCTTCGTACGCTGCAGGTCGACG	*RNR2* disruption
*RNR2*DRv	GATTGAAGAGACTGCGTAAAAAGAAATATATAGA GAGATACTCGATATCATCGATGAATTCGAGC	*RNR2* disruption
*RNR2*PstICFw	GCCGGCTGCAGCCCCGTTGCCACAGAGACCAC	*RNR2* cloning
*RNR2*PstICRv	GCCGGCTGCAGCTGCACCGAGGCCGTTGAGATC	*RNR2* cloning
*RNR2*L362VFw	TCGATTTCATGGAAAACATCTCCgTaGCCGGTAAGA CCAACTTCTTCG	*RNR2* mutagenesis
*RNR2L362*VRv	CGAAGAAGTTGGTCTTACCGGCtAcGGAGATGTTTT CCATGAAATCGA	*RNR2* mutagenesis

Table S1. Primers used. Nucleotide changes are in lowercase.
